# Antibiotic Usage in Patients Having Undergone Caesarean Section: A Three-Level Study in Benin

**DOI:** 10.3390/antibiotics11050617

**Published:** 2022-05-04

**Authors:** Angèle Modupè Dohou, Valentina Oana Buda, Loconon Achille Yemoa, Severin Anagonou, Françoise Van Bambeke, Thierry Van Hees, Francis Moïse Dossou, Olivia Dalleur

**Affiliations:** 1 Louvain Drug Research Institute, Université Catholique de Louvain, Avenue Emmanuel Mounier 73, 1200 Brussels, Belgium; francoise.vanbambeke@uclouvain.be (F.V.B.); olivia.dalleur@uclouvain.be (O.D.); 2 Faculté des Sciences de la Santé, Université d’Abomey Calavi, Cotonou 01 BP 188, Benin; ayemoa@yahoo.fr (L.A.Y.); anagonou_severin@yahoo.fr (S.A.); dosfm@yahoo.fr (F.M.D.); 3 Faculty of Pharmacy, “Victor Babes” University of Medicine and Pharmacy, Eftimie Murgu Square, No. 2, 300041 Timisoara, Romania; buda.valentina.oana@gmail.com; 4 Center for Interdisciplinary Research on Medicines, Université de Liège, Place du 20 Août 7, 4000 Liège, Belgium; thierry_vanhees@outlook.com; 5 Service de Pharmacie Clinique, Cliniques Universitaires Saint-Luc, Avenue Hippocrate 10, 1200 Brussels, Belgium

**Keywords:** caesarean section, antenatal antibiotic use, antibiotic prophylaxis, postpartum antibiotic use, Benin

## Abstract

The intense use and misuse of antibiotics is undoubtedly the main factor associated with the high numbers of antibiotic-resistant pathogenic and commensal bacteria worldwide. In low-income countries, this misuse and overuse is widespread, with great consequences at the personal and global levels. In the context of user fee exemptions in caesarean sections, we performed a descriptive study in women to assess the use of antibiotics on three levels—antenatal, during caesarean section, and postpartum—in four Beninese hospitals. Out of the 141 women included, 56.7% were using antibiotics. More than the half (71.3%) were taking more than one antibiotic, either for a long time or in acute treatment. In prophylaxis, the timing, dose, and duration of administration were not correctly achieved. Only 31.2% of women received optimal antibiotic prophylaxis. Various antibiotics including broad-spectrum molecules were used in the patients after caesarean section. The use of antibiotics was improper on the three levels studied. The high rate of self-administered antibiotics, the poor achievement of antibiotic prophylaxis, and the postpartum overuse of antibiotics showed a poor quality of care provided in pregnancy. A national policy is essential to improve the use of antibiotics by the general public as well as by professionals.

## 1. Introduction

The intense use and misuse of antibiotics is undoubtedly the main factor associated with the high numbers of antibiotic-resistant pathogenic and commensal bacteria worldwide [1]. The resistance of bacteria to antimicrobials has become one of the most serious global public health threats in this century, with the greatest impact in developing countries, where the burden of infectious diseases is much higher [2,3]. The World Health Organization (WHO) says that steps can be taken at all levels of society to reduce the impact and limit the spread of resistance [4], by reducing the incidence of infectious diseases, increasing knowledge and awareness, and promoting rational use of antimicrobials, among other measures [5]. This kind of action can be effective when data on antibiotic use and antimicrobial resistance evolution are available.

Antibiotics in pregnancy are used to treat the infections encountered during this physiological state, such as urinary tract infections, pyelonephritis, sexually transmitted infections, and upper respiratory tract infections [6]. A limited number of antibiotics are considered safe and effective in pregnancy, and their use has to be based on a risk-versus-benefit decision [7]. In low-income countries such as Benin, the antibiotics used come from medical prescriptions or direct purchase from a pharmacy. Studies have shown that the poor quality of the prescription of antibiotics in patients [8], along with the possibility of purchasing antibiotics without medical prescription, has significant consequences in terms of antimicrobial resistance [9]. Antibiotics are also used when women have to undergo caesarean section for prophylaxis. Antibiotic prophylaxis is defined as “a brief course of an antimicrobial agent initiated before an operation begins in order to reduce intraoperative microbial contamination to a level that will not overwhelm host defence and result in infection” [10]. Conventionally, antibiotic prophylaxis must meet five criteria: indication, choice of molecule, dose, timing, and duration [11]. The objectives of antibiotic prophylaxis are, firstly, to achieve sufficient antibiotic tissue concentration before possible bacterial wound contamination and, secondly, to ensure adequate levels throughout the operative procedure to prevent subsequent bacterial growth [12]. Antibiotic prophylaxis is shown to lower the incidence of infection after surgeries [13]. Although antibiotic prophylaxis recommendations are known worldwide, studies have shown that surgical teams have low adherence to respecting those recommendations [14]. Adherence to recommendations in Benin is unknown.

Caesarean section has been known to increase the rates of maternal infectious morbidities fivefold compared to vaginal delivery. Antibiotic prophylaxis can play a major role in improving caesarean-section-associated risks of infections, such as endometritis, surgical site infections, and urinary tract infections [15,16]. The practice of antibiotic prophylaxis according to the guidelines decreases the poor outcome consequences of caesarean section, such as long hospital stays, systematic antibiotherapy leading to overuse or inappropriate use of antibiotics and the development of antimicrobial resistance, additional costs, and psychological and social impacts [16].

In 2009, the Government of Benin set up a policy of user fee exemptions for caesarean sections, and an agency was created to manage the policy. A free kit containing the required medicines—including antibiotics (injectable: 1 g ampicillin, 500 mg/100 mL metronidazole, and 80 mg/2 mL gentamicin for prophylaxis; and in oral forms: 500 mg amoxicillin and 500 mg metronidazole tablets for postpartum)—and materials needed for the procedure is available.

Unlike in high-income countries, data on the use of antibiotics are scarce in low-income countries, where hygiene conditions are poor and antimicrobial resistance is of great concern [17]. In Benin, several studies have reported an increasing evolution of antimicrobial resistance [18,19,20], but there are no updated data on antibiotic use. Moreover, the use of antibiotics in caesarean section has not been described since the implementation of the caesarean section user fee exemption in Benin. To make data available in Benin, we aimed to document the use of antibiotics in a population of women undergoing caesarean section in four hospitals in Benin, by conducting a descriptive study on three levels: antenatal (a patient’s medication history in terms of antibiotics used prior to hospital admission), an audit of surgical antibiotic prophylaxis for caesarean section, and the postpartum use of antibiotics.

## 2. Results

In total, 141 women were included in our study. The patients’ age range was 17–42 years, with a median of 28 years; 81.60% of the cases (*n* = 115) were emergency, and 27.60% (*n* = 39) were referred from another hospital. The median duration of the intervention was 39.20 min, ranging between 13 and 75 min. Among the patients, 8.5% (*n* = 12) had a chronic disease (e.g., diabetes, sickle cell anemia, cardiovascular or hepatic disease). Patients’ demographic and clinical characteristics are displayed in Table 1.

## 2.1. Antenatal Antibiotic Use

We interviewed 141 patients about their medication with antibiotics prior to their admission to hospital; 80 (56.70%) of them declared they were using antibiotics. A total of 23 (28.70%) were taking one antibiotic, and more than half (57 of 80; 71.30%) were taking 2–5 antibiotics, either for a long time (more than 15 days) or in acute treatment. The most frequent antibiotic used was amoxicillin in monotherapy (*n* = 16, 20.0%), or combined with metronidazole (*n* = 14, 17.50%) or ciprofloxacin (*n* = 12, 15.0%). Other antibiotics (e.g., ampicillin, cloxacillin, erythromycin, co-trimoxazole) were used by 38 of them (47.50%).

Nearly half of the patients (*n* = 38, 47.50%) were taking antibiotics under their own initiative. Almost the same proportion (*n* = 36, 45.0%) indicated that the antibiotics were prescribed by a healthcare professional, but more than half could not give the precise reason for the prescription. The remaining women (*n* = 6, 7.50%) were using antibiotics based on the recommendations of their relatives. In the case of their own initiative, patients declared having the information from a previous medical prescription. This could be due to the fact that the prescribers did not educate patients after prescription to avoid inappropriate use of antibiotics.

Among the indications (e.g., malaria, fever, pain, infectious diseases), stomach trouble was the most frequent indication declared by the patients (*n* = 13, 16.30%). The patients did not bring their medicines to the hospital.

The overview of the use of antibiotics prior to hospital admission is reported in Table 2.

## 2.2. Antibiotic Prophylaxis in Caesarean Section

The antibiotic prophylaxis practices were assessed by the five conventional criteria (indication, choice of molecule, dose administered, timing, and duration of administration) in the four hospitals. Four of the five criteria were less appropriately achieved. Only 31.2% (44/141) of women received optimal antibiotic prophylaxis, i.e., correct antibiotic prophylaxis according to the five criteria. Sixteen different antibiotic regimens were observed; three of them contained the three molecules of antibiotics from the kit, and were administered in 17.0% of patients (24/141). The most frequent regimen administered was amoxicillin + clavulanic acid combined with metronidazole in 27.0% (38/141), which represented 70.4% (38/54) of patients who received the antibiotics not included in the kit.

**Figure 1:**
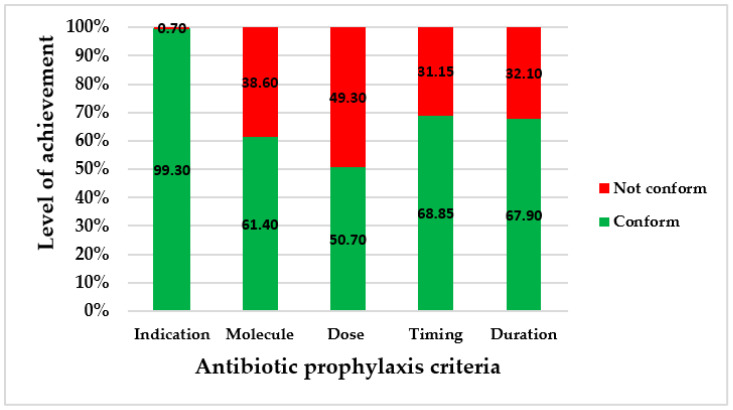
Levels of achievement of conventional criteria for antibiotic prophylaxis.

From the data collected in the four hospitals, the levels of achievement are represented in Figure 1.

## 2.3. Postpartum Antibiotic Use

After caesarean section, all 141 patients received oral antibiotics for 5 to 10 days, depending on the prescriber. The oral antibiotics (amoxicillin + metronidazole) included in the kit were used by 48.2% (68/141) of the women. The others 57.7% (73/141), in addition to metronidazole, received out-of-kit and broad-spectrum antibiotics, such as amoxicillin + clavulanic acid in 43.3% (61/141), lincomycin in 7.8% (11/141), and cefixime in 0.7% (1/141). The broad-spectrum antibiotics used were variable from one hospital to another. Each hospital used the same broad-spectrum antibiotic in all of their patients. We did not record any diagnosis for the use of postpartum antibiotic therapy.

## 3. Discussion

Our study describes the perinatal use of antibiotics in women who underwent caesarean section in four different hospitals in Benin.

Firstly, the medication history showed the use of antibiotics prior to hospital admission in 56.7% of the women. This rate was higher than that reported in the Democratic Republic of Congo in 2013 (12.3% of patients admitted in emergency) [21], and represents an enormous concern in developing countries. Merrett et al. stated that self-medication with antibiotics in low- and middle-income countries is widespread, and leads to overuse and misuse of antibiotics, with consequences on the personal and global levels [22]. Some of the patients in our study were taking antibiotics based on medical prescription; others did so as self-medication, and declared that this habit was based on a former healthcare professional’s prescription, or on relatives’ advice. The large proportion of self-medication noted could be due to the fact that patients could buy antibiotics without medical prescription at the time we collected the data. Although we did not collect data on the women’s knowledge and education levels, we know from the literature that the low levels of those parameters negatively impact behaviors regarding medication [23], and that women in Benin are less educated than men [24]. Socioeconomic and behavioral factors (e.g., out-of-pocket payments, absence of health insurance, level of education, and culture) were reported to explain the self-medication [3,25,26]. From the analysis of our data, we can hypothesize a lack of useful required information provided by healthcare professionals in outpatient care. Indeed, the patients could not explain their precise reasons for taking antibiotics. Additionally, some of the pregnant women had used ciprofloxacin, while the use of fluoroquinolones should be avoided unless the benefits outweigh the risks [7]. This shows the poor quality of healthcare delivered in our country, as reported in the second edition of “Disease Control Priorities in Developing Countries”, with a need for improvement [27]. One of the most important ways to improve health in developing countries is by educating citizens. Educating people enables them to increase their health literacy, take preventive healthcare measures, avoid riskier health behaviors, and demand better quality healthcare services [27]. Considering the enormous threat that antimicrobial resistance represents to public health, and the place of mothers in families’ health (in most households, women are the managers of their families’ healthcare needs) [28], educating pregnant women will help not only them and their babies, but also other members of their families, to avoid inappropriate use of antibiotics.

Secondly, our study identified improper practices of antibiotic prophylaxis in caesarean section. Apart from the indication that was conformed to by 99% of the patients, either in the operating room or in the recovery room, the four other criteria (molecule, dose, timing, and duration) were not appropriately applied in at least 30% of cases. A proportion of 49.30% of patients did not receive the correct dose of antibiotic. The criterion of the dose administered was the least appropriately achieved.

Under one-third of women (31.20%, 44/141) received antibiotic prophylaxis with the five conventional criteria correctly properly achieved. The aim is that antibiotic prophylaxis should be correctly achieved in 100% of cases. However, this compliance rate was higher than those reported in a Jordanian study (2.70%) [29] and in a Greek study (0%) [30]. This finding can be explained by the availability and use of the antibiotics in the kits in some of the hospitals studied. This proportion has to be improved on, especially since it has been reported that the use of the kits significantly enhances the national guidelines on the surgical application of antibiotic prophylaxis [31].

Although all of the patients received a prophylaxis, non-conformities were observed, such as for the timing in 31.10%, versus 0% in a Greek study [30] and 11.60% in a Sudanese study [16]; the dose administered, with 49.30% of women under-dosed versus 37.50% in a Jordanian study [29]; and the duration of administration, exceeded in 32.10% of patients, in contrast to 88.20% reported in a Jordanian study and 97% in a Sudanese study [10,29]. The consequences of these non-conformities are numerous. The delay in timing reduces the patient’s protection during surgery, and can lead to the occurrence of postoperative infections. The optimization of the initial dose of antibiotics in prophylaxis is important in order to establish an inhibitory concentration of the drug in the serum and tissues by the time the skin is incised [11]. Concerning the under-dosing, it results in low plasma concentrations of antibiotics, leading to a risk of surgical site infections or endometritis [11]. The long course of intravenous administration contributed to an overuse of antibiotics for prophylaxis. A continued course of antibiotic prophylaxis pressurizes the nurses, and affects their workload [12]. The delay of timing and long duration of prophylaxis induce financial burden for patients, because postoperative expenses due to infections are not taken into account in the Benin user fee exemption policy, and patients must pay for the additional needed and prescribed antibiotics outside the kit in order to complete the antibiotic prophylaxis duration. The WHO recommends a single dose of antibiotic [11], as concluded in a randomized study performed in other low-income countries [12,32]. This finding appeared paradoxical in this study, meaning that antibiotic prophylaxis was not administered with good timing and dose, but beyond the recommended duration. This attitude could be due to the fear of post-caesarean infections caused by the particular problems that low-income countries such as Benin experience, as the women accumulate all factors causing the high rate of maternal mortality, such as suboptimal health status, nutritional deficiencies, lack of hygiene, scarce resources, and inadequate knowledge [12]. This fear thus induces the use of broad-spectrum antibiotics. In fact, despite the availability of antibiotics in the kit, amoxicillin + clavulanic acid was used in 26.9% of cases, ceftriaxone in 3.5%, and ceftriaxone + sulbactam in 0.7%. This finding is consistent with those reported in a Sudanese hospital, where broad-spectrum antibiotics were used in 56.3% of cases in spite of the availability of the recommended antibiotic (cefazolin) [10]. This point highlights a veritable problem of adherence to the guidelines by the surgical teams, and results in the irrational use of antibiotics and increases the occurrence of antimicrobial resistance [10].

In addition to the cost the prescription of out-of-kit antibiotics represents (three boxes of amoxicillin + clavulanic acid cost USD 10.57–16.95, versus free antibiotics in the kit), patients have to face the non-availability of these prescribed antibiotics in the hospital pharmacies. Thus, patients were often obliged to buy antibiotics from private pharmacies. In view of the fact that most of the women were presented in an emergency situation (81.6% of cases) and/or often were referred from peripheral health centers, the availability of the required antibiotics in the hospital pharmacy becomes essential. This will be a national challenge for Benin in the coming years, as has been reported in other low-income countries, such as Mozambique [12].

Finally, in the postpartum use, antibiotics were available in Benin’s free kit by providing oral antibiotics (amoxicillin + metronidazole). According to the WHO, the antibiotics recommended are the combination of clindamycin and gentamycin as first-line treatment for postpartum endometritis [33]. In the case of the women included in our study, no diagnosis of endometritis or other infection was made, but broad-spectrum antibiotics (e.g., amoxicillin + clavulanic acid, lincomycin, cefixime) were used in several cases. However, although the antibiotics provided in the kit are intended for postpartum endometritis, it is important to note that they are different from the one recommended by the WHO (clindamycin), which is not currently available in Benin.

The global analysis of the use of antibiotics in the patients who underwent caesarean section in the four hospitals chosen, following the three levels of the healthcare system in Benin, highlighted either inappropriate use, misuse, or overuse of antibiotics. Although practices were similar, showing a local consensus within each hospital, the healthcare professionals in high-level hospitals were less adherent to the kit. In sum, we found a poor quality of care provided for pregnancy, since quality of care in obstetrics is a continuum that spans the pre-pregnancy, pregnancy, and postpartum periods. Regarding the context of the increased spread of bacterial resistance, rational use of antibiotics would benefit public health, as well as the patients’ and the hospitals’ finances.

To improve the use of antibiotics in Benin, various levels of the healthcare system have to be taken into account. First of all, patients could be educated in hospitals or through mass media by providing information on antibiotics (e.g., when to use, resistance problems, and prevention measures to avoid infections). Secondly, it is important to raise the level of knowledge of the healthcare professionals in terms of the prescription of antibiotics, to conduct audits and feedback in hospitals, and to provide local and consensual guidelines for the use of antibiotics. Setting up a multidisciplinary team in hospitals can also help to improve the use of antibiotics. Thirdly, at the central level, a national policy is essential to coordinate activities by providing data on antibiotics consumption and antimicrobial resistance, along with directives and strategies to improve utilization.

To the best of our knowledge, our study is the first to analyze the use of antibiotics before, during, and after caesarean section in Benin and in various types of hospitals. Observations were conducted 24 h a day, 7 days a week. However, this study has some limitations: All of the observed hospitals were from the southern part of the country; thus, they are not entirely representative. The sample size was relatively small. In addition, there was no assessment of factors associated with inappropriate use of antibiotics. Nevertheless, we are confident that our findings will help to raise knowledge about the use of antibiotics in Benin, and help to perform this kind of study in other areas of the country.

## 4. Materials and Methods

In obstetrical wards of 4 hospitals located in the south of Benin, we performed a three-level study on antibiotic use in patients who were undergoing caesarean section. Patients were approached in the operating theatre when medical staff were preparing the intervention. The study was briefly explained in the patient’s language. The sampling followed the inclusion and exclusion criteria as described in the document entitled “L’audit clinique: bases méthodologiques de l’évaluation des pratiques professionnelles” [34], with the objective of having at least 100 cases of caesarean section observed during the study period for this first insight into antibiotic use in Benin.

We recorded patients’ medication history by interviewing the patients on their habits of using antibiotics prior to admission, conducting a prospective observation of the antibiotic prophylaxis practices, and determining postpartum antibiotic use from medical charts. The four hospitals have different characteristics, but they are all approved to practice free caesarean section. They included one teaching referral hospital (hosp1: 254 beds), two teaching zonal hospitals (hosp2: 101 beds and hosp3: 116 beds), and one confessional hospital (hosp4: 81 beds). Each hospital included in this study provided written authorization to perform all of the steps of the study. Over a period of 1 month (November to December 2016), observers’ teams collected data from patients undergoing caesarean section with class 2 surgical wound contamination (clean–contaminated wound, any infection diagnosed before caesarean section), using two survey forms (Appendix A.1 and Appendix A.2).’’

Form No. 1 was used to collect data on demographic characteristics (e.g., name, age) and patients’ medication history (e.g., oral antibiotics used, indication for utilization, author of the prescription, regimen of administration). The patients’ medication history was recorded after the intervention in the hospitalization rooms.

Form No. 2 is divided into two parts: The first part helped to collect data on demographics (e.g., name, age)—in order to be sure that we were collecting data from the same patient as the previous form—intervention date in the operating theatre, dates of admission and intervention, type of procedure (urgent or elective), and antibiotics used for prophylaxis (molecule, dose, timing, and duration). This part of the form was adapted from those used by the WHO and the Scientific Institute of Public Health, Belgium [35,36].

In the second part of form No. 2, we collected follow-up data during hospitalization from medical charts, including intravenous antibiotics used after caesarean section (name, dose, posology, and duration), and the postpartum use of oral antibiotics i.e., after delivery (name, dose, posology, and duration).

Observers’ teams consisted of four pairs of students of the health science faculty. After a training session with the main researcher, the four pairs tested the applicability of the forms in two other hospitals that were not included in the present study. After the test, a debriefing took place, and some questions were reviewed. During data collection, one observer included patients after obtaining their consent, and recorded data in the operating theatre during the caesarean section procedure using the first part of form No. 2. The second observer recorded data in the hospitalization rooms until the end of the intravenous administration in the second part of form No. 2. Patients were included alternatively in different periods of the day (intervention in the morning, afternoon, evening, and night) in order to see whether practices changed from time to time.

The practices of antibiotic prophylaxis were assessed based on the following five conventional criteria [11]:

Indication: In caesarean section, antibiotic prophylaxis was always considered to be indicated.

Choice of antibiotic: The conventional recommended antibiotic is cefazolin. In Benin, cefazolin is not available. The Benin caesarean kit instead contains ampicillin, metronidazole, and/or gentamicin. The choice of antibiotic was considered to be correct if the antibiotics from the kit were used.

Timing of administration: Guidelines recommend administration of the first preoperative dose 30–60 min before incision.

Accuracy of the first preoperative dose(s): Double the usual adult dose of an antibiotic is recommended for caesarean section. Any necessary subsequent doses are administered at the usual adult dose.

Duration of prophylaxis: In caesarean section, antibiotic prophylaxis can be administered in a single dose, or for a period of 24 h maximum.

Data were analyzed using IBM Corp (released 2016; IBM SPSS Statistics for Windows, Version 24.0. Armonk, NY, USA: IBM Corp). Descriptive statistics presented continuous variables using medians (with 25–75th percentiles), because they were not normally distributed, and categorical variables are presented as numbers and percentages.

## 5. Conclusions

Caesarean section is a life-saving intervention. The conventional recommended antibiotic use is for prophylaxis, which is an incontestable parameter to ensure quality of life for patients. Despite the availability of a free kit containing the antibiotics required, antibiotic prophylaxis was poorly achieved in Benin. In the use of antibiotics prior to hospital admission, patients were insufficiently informed for the precise reason of their use if they had a medical prescription. In the postpartum period, there was no diagnosis of postoperative infection that could require the use of antibiotics. In sum, self-administered antibiotics require particular attention in order to reduce their rate and long-term consequences. Various measures should be embedded in a national policy to ameliorate the use of antibiotics by the general public and by professionals in Benin.

## Figures and Tables

**Table 1:** Patients’ demographic and clinical characteristics.

Hospital Studied	Parameters	Hosp1	Hosp2	Hosp3	Hosp4	Total
Demographic characteristics	Number of patients, *n* (%)	50 (35.50%)	49 (34.70%)	22 (15.60%)	20 (14.20%)	141 (100%)
	Median age (years) ± standard deviation	28.6 ± 5	27.4 ± 5.9	27.9 ± 4.4	28.1 ± 3.8	28 ± 4.8
	Range	(20–42)	(17–41)	(21–37)	(22–37)	(17–42)
Clinical characteristics	Emergency cases, *n* (%)	35 (70.0%)	44 (89.80%)	21 (95.50%)	15 (75.0%)	115 (81.60%)
	Referred cases, *n* (%)	16 (32.0%)	16 (32.60%)	6 (27.30%)	1 (5.0%)	39 (27.60%)
	Median intervention duration (minutes) ± standard deviation	48.0 ± 14.8	37.40 ± 10.1	35.70 ± 10.1	35.30 ± 13.0	39.20 ± 12
	Range	(24–85)	(13–75)	(22–53)	(19–69)	(13–75)

**Table 2:** Repartition of the utilization of antibiotics prior to admission.

Patients (*N* = 141, 100%)	Characteristics *n* (%)
**Patients who used antibiotics**	80 (56.70%)
**Number of antibiotics used** 1	23 (28.70%)
2 to 5	57 (71.30%)
**Antibiotics used**	
Amoxicillin	16 (20.0%)
Amoxicillin + metronidazole	14 (17.50%)
Amoxicillin + ciprofloxacin	12 (15.0%)
Others (ampicillin, cloxacillin, erythromycin, co-trimoxazole)	38 (47.50%)
**Type of use**	
Self-administration	38 (47.50%)
Medical prescription	36 (45.0%)
Relatives’ advice	6 (7.50%)

## Data Availability

Not applicable.

## Appendix A

### Appendix A.1. Antibiotics Use in Patients Underwent Caesarean Section: Medication History

Date of collect:.../…./…..

Patient identity: ………………………………………… Patient age: ………………

Hospital: ……………………….

**Do you use to take antibiotics?** Yes □ No □

**Which antibiotic do you use to take?** Ampicillin □ Amoxicillin □ Ciprofloxacin □ Cotrimoxazole □ Erythromycin □ Metronidazole □ Doxycycline □ Other (write the name): ………………….…………………………………………………………………
**Who prescribed this antibiotic for you?** ……………………………………………………………
**How long have you been taking it?** ………………………………… (days, months, years)
**How often do you take it?** Usually □ Often □ Rarely □ If needed □ **For which disease?**:……………………………………………………………………………………………
**Are you taking this antibiotic during your hospitalization?** Yes □ No □

### Appendix A.2. Use of Antibiotics in Prophylaxis and Post-Partum in Caesarean Section

Date of collection…………………………… sheet N………..

Hospital:…………………….

**Nota Bene**

(a) Use one form per patient
(b) Pay attention on inclusion criteria.
  I **Patient data**
  Name and first names: ……………………………………………………………………………..
  (Will not be published)
  Birthday or age of patient: ……………………………………………………………………….
  Chronic disease/Comorbidity
  □ Diabetes (type ….) □ Cardiovascular disease: ………………..
  □ Liver disease: ………………………… □ other: ………………………………..
  Transferred from other hospital: □ Yes □ No
  Timing of intervention after transfer: …… days …… hours
  II **Surgical intervention**
  Date of intervention: ……/……/………
  Type of wound contamination: ………………………. (Class 2)
  Duration of intervention: ……………hours ……..… minutes
  Type of intervention □ Elective □ Emergency
  **Part 1**
  III **Antibiotic prophylaxis**
    Is antibiotic prophylaxis realized Yes □ No □
          Oral route □ Intra-venous □
    Timing of administration before incision □ after incision □
    Time of antibiotic’s administration
          If before incision: --- days --- hours --- minutes
          If after incision: --- days ---hours --- minutes
    Names of antibiotics administered
    (1) ……………………………………………………………………………………
    (2) …………………………………………………………………………………..
    (3) ……………………………………………………………………………………
    Dose of antibiotics administered:
    (1) …………………………………………………………………………………
    (2) …………………………………………………………………………………
    (3) …………………………………………………………………………………
    (Re) Administration of antibiotics during intervention
          □ No □ Yes Time: …………hours … minutes.
    Administration of antibiotics in wake up room
          □ Yes □ No
    If yes, name of antibiotics, dose, route of administration and duration:
    (1) ……………………………………………………………………………………………………
    (2) ……………………………………………………………………………………………………
    (3) ………………………………………………………………………………………………………
    **Part 2**
    Administration of IV antibiotics in hospitalization room: (Name, dose, and duration)
    (1) ………………………………………………………………………………………………………
    (2) ……………………………………………………………………………………………………
    (3) ………………………………………………………………………………………………………
  IV **Oral antibiotic in post-partum (Name, dose, and duration)**
    (1) ………………………………………………………………………………………………………
    (2) ……………………………………………………………………………………………………
    (3) ………………………………………………………………………
